# State of the neoadjuvant therapy for glioblastoma multiforme—Where do we stand?

**DOI:** 10.1093/noajnl/vdae028

**Published:** 2024-03-05

**Authors:** Naeim Nabian, Reza Ghalehtaki, Mehdi Zeinalizadeh, Carmen Balaña, Paola Anna Jablonska

**Affiliations:** Radiation Oncology Research Center, Cancer Research Institute, Tehran University of Medical Sciences, Tehran, Iran; Department of Radiation Oncology, Cancer Institute, Imam Khomeini Hospital Complex, Tehran University of Medical Sciences, Tehran, Iran; Radiation Oncology Research Center, Cancer Research Institute, Tehran University of Medical Sciences, Tehran, Iran; Department of Radiation Oncology, Cancer Institute, Imam Khomeini Hospital Complex, Tehran University of Medical Sciences, Tehran, Iran; Department of Neurosurgery, Tehran University of Medical Sciences, Tehran, Iran; B.ARGO (Badalona Applied Research Group of Oncology) Medical Oncology Department, Catalan Institute of Oncology Badalona, Badalona, Spain; Radiation Oncology Department, Clinica Universidad de Navarra, Pamplona, Spain

**Keywords:** adjuvant, chemotherapy, glioblastoma, neoadjuvant therapy, neurosurgery, radiotherapy

## Abstract

Glioblastoma multiforme (GBM) is the most common malignant primary brain tumor in adults. Despite several investigations in this field, maximal safe resection followed by chemoradiotherapy and adjuvant temozolomide with or without tumor-treating fields remains the standard of care with poor survival outcomes. Many endeavors have failed to make a dramatic change in the outcomes of GBM patients. This study aimed to review the available strategies for newly diagnosed GBM in the neoadjuvant setting, which have been mainly neglected in contrast to other solid tumors.

Glioblastoma multiforme (GBM) is the most common malignant primary tumor of the central nervous system in adults.^1,2^ Its annual age-adjusted incidence ranges between 0.59 and 5 per 100,000 population worldwide.^3^ GBM is considered one of the most aggressive, invasive, and undifferentiated types of brain cancer. The 5-year survival rate is only 5.6% which represents the aggressive manner of this pathology.^1^

Maximal safe resection followed by concurrent chemoradiotherapy and adjuvant temozolomide (TMZ) for 6 cycles form the current standard of care for GBM. This combination of postoperative therapies is collectively known as the “Stupp protocol.”^4^ Surgery, preferably gross total resection (GTR) or even a supratotal resection due to its positive impact on the outcomes, is the gold standard surgical procedure for GBM.^5,6^ It confirms the diagnosis, provides tissue for molecular analysis, and improving the symptoms by quickly reducing the mass effect. Unfortunately, GTR might not be feasible depending on the location or the technical limitations, and partial resection or even biopsy may be offered to some patients.^7^ Even if the GTR is possible, the infiltrative nature of GBM makes the surgery per se insufficient for achieving a definitive cure.^8^ In fact, without multimodality adjuvant treatment, the overall survival (OS) is very low.^9^

Radiation therapy (RT) eliminates microscopic residual disease at the primary site in the vicinity of the grossly resected lesion or the unresected macroscopic residual tumor so that it can maximize the chances of local control.^10^ However, RT is limited by the surrounding anatomical structures with a known dose tolerance.^11^ RT is delivered using one of the 3D conformal or intensity-modulated radiotherapy (IMRT) or volumetric modulated arc therapy techniques to a standard dose of 60Gy in 30 fractions, with hypofractionated regimens reserved for elderly or fragile patients (40Gy/15 or 20Gy/5).^12,13^

The standard chemotherapy includes oral TMZ, given concurrently with RT at a daily dose of 75 mg/m^2^ from the first to the last day of RT, followed by an adjuvant dose of 150–200 mg/m^2^ for 5 days up to 6 cycles which are repeated every 4 weeks. The prospective studies following Stupp failed to show added benefit from extension of TMZ cycles to one year or 12 cycles.^14^ In addition, drug development studies have been largely unsuccessful after TMZ due to the presence of the blood-brain barrier (BBB), which prevents most cytotoxic agents from accumulating in the brain.^15^

After the landmark EF-14 trial, tumor treating field (TTF) has also been approved by the United States Food and Drug Association (US FDA) for the postoperative setting after CRT and concomitant by the maintenance TMZ.^16^ By delivering alternating low-intensity electric fields, TTF interferes with the mitotic activity of GBM cells and confers progression-free survival (PFS) and overall survival (OS) benefits. However, its use has remained very low even in academic referral sites for neuro-oncology practice.^17^

Following the establishment of the standard of care in GBM, there have been some investigational modifications to improve the outcomes in patients. For example, the addition of lomustine to the RT plus TMZ in methylated MGMT patients was a successful maneuver in the phase III CeTeG/NOA-09 trial.^18^ However, due to the small sample size and other limitations, this trial was not been able to change the standard of care so far.

Other than RT, TMZ, and TTF, adjunctive treatments suggested for GBM have been tested or proposed that include targeted therapies and immunotherapy (IO), Boron neutron capture therapy (BNCT) and epigenetic therapy, oncolytic virus therapy (OV), and gene therapy. None of these treatments has become the standard of care for the primary GBM treatment as yet. Many of these adjunctive therapies are still in the preclinical state, some were tested in the clinical settings and were ineffective or needed more evidence to integrate with the standard of care.

BNCT is a modality for biochemical adjunctive therapies in which boron is delivered using a selective vector to the neoplastic tissue. Because of the damaged BBB or augmented expression of the amino acid transporters on the tumor cell surface, boron only concentrates in the tumor. Then, neutron particles are irradiated from an external device, and then boron and neutron react in the tumor cell. This process theoretically provides normal tissue-sparing and high-dose radiation delivery. Efforts in BNCT of brain tumors started in 2002, and available results demonstrate the beneficial effect of such therapy on the survival of patients with both recurrent and newly diagnosed glioblastomas.^19^

Oncolytic virotherapy (OV), a subgroup of immunotherapy (IO), is another landscape studied in clinical trials with overall safety but variable efficacy. OV is based on converting the immunogenically “cold” brain tumors to “hot” ones that stimulate the immune system. There are two types of oncolytic viruses. The first type, called replication-competent, includes the viruses that invade the tumor cell and start replication until cell death. Then, the replicated viruses can invade other tumor cells and evoke an immune response due to the release of massive tumor cell antigens. The second type, selectively replication-competent, includes viruses used as vectors to apply gene therapy.^20^ So far, only Teserpaturev/G47 Delta, a replication-competent recombinant herpes simplex virus, has received conditional and time-limited approval from the Japanese authorities for use in gliomas based on the GD-01 phase II trial.^21^

Another investigational treatment for GBM is gene therapy, defined as inserting a mutated gene or transcript in the tumor cells by way of mRNAs or in combination with nanoparticles.^22^ Gene therapies that impact GBM cells with specific mutations seem more popular in ongoing trials.

One treatment approach that is so attractive in other solid tumors is targeted therapies using monoclonal antibodies or small molecule tyrosine kinase inhibitors (TKI). Numerous medications have been tested in this regard, and all have been unsuccessful except bevacizumab and BRAF inhibitors, which showed some activity in phase II but unfortunately not in phase III trials.^23,24^

The latest trendy approach for the treatment of GBM has been the use of an immune checkpoint inhibitor (ICI). ICIs upregulate immune response and reveal the concealed tumor cell to the immune system. So, the body itself can eliminate it. In practice, however, despite the positive results of phase I Checkmate-143, which showed safety and some promising findings,^25^ the phase III Checkmate-498 and Checkmate-548 trials disastrously failed. In Checkmate-498 in exclusively unmethylated MGMT patients, nivolumab plus RT was significantly inferior to TMZ and RT in terms of OS.^26^ Seemingly, in Checkmate-548 in methylated MGMT patients, the addition of nivolumab to RT plus TMZ could not increase OS and PFS despite increased toxicity.^27^ The failure of ICIs, which is attributable to the immunologic coldness of brain tumors, have made investigators combine ICI with OV-based therapies, as described above, to see whether it can improve results. CAPTIVE phase I/II trial tested a single intratumoral injection of an oncovirus called DNX-2401 combined with intravenous multiple doses of pembrolizumab in recurrent GBM with a promising 1-year OS rate of about 52%.^28^ Ongoing trials are in way testing if this approach has efficacy in the primary GBM as well.

Despite all the mentioned maneuvers, median overall survival is slightly more than one year from diagnosis, so that even in the most favorable situations, the majority of patients do not survive beyond 2 years.^4^ An area that has been less investigated and merits more attention is neoadjuvant therapy (NAT). NAT is defined as applying another effective treatment before the main local treatment, which is surgery or RT in most cancers. NAT has been shown to improve clinical outcomes in many solid malignancies.^29^ So, in the following sections, we review the current neoadjuvant treatment strategies for newly diagnosed and recurrent GBM. We have been focusing on their feasibility, safety, and efficacy and describing future directions in this field.

## Methods

The characteristics and results of original retrospective and prospective studies investigating the efficacy of neoadjuvant therapies for patients with resectable or unresectable GBM (primary or recurrent) were reviewed. It was necessary to report the OS and PFS specifically for GBM.^30–32^ Also, studies conducted before the approval of chemoradiation as the standard of care for newly diagnosed GBM in 2005 were not included.^4^

### Neoadjuvant Therapy for Newly Diagnosed GBM

NAT has been established as a standard of care in many primary solid malignancies. For example, in rectal cancer where the bony pelvis limits surgical resection, it has been proved that pre-operative or NAT confers lower toxicity and better sphincter preservation than post-op or adjuvant therapy.^33^ The German Rectal Cancer Study group’s trial was the most important study that made the pre-op CRT vs. post-op CRT the standard of care in rectal cancer.^34^ With preoperative CRT compared to the postoperative treatment, there was a statistically significant decrease in local failure and a significant decrease in acute toxicity and late complications. Nevertheless, even with an 11-year follow-up, there was still no effect on OS or distant metastases.^35^ We brought the rectal cancer example to emphasize the possible role of NAT in the milieu of a limited surgical resection. This phenomenon is shared between rectal cancer and GBM.

There are theoretical benefits to NAT that are evident in other solid tumors such as soft tissue sarcoma, breast, esophagus, or rectal cancer, with implications in GBM. The use of neoadjuvant treatment has the following justifications: (a) in case of response to NAT, the resultant down-sizing of the tumor would make the surgery easier with less damage to normal surrounding tissues.^36^ In case of GBM, this can result in less extensive surgery with subsequent better outcomes; (b) in terms of neoadjuvant RT, we can see the gross target volume so the target delineation can be done more confidently, the target volume is smaller due to absence of a surgical bed, better oxygenation of the unmanipulated tissues requiring less dose to exert the same effects, and removal of much of irradiated tissue during surgery that would decrease the chance of radionecrosis and subsequently less toxicity of RT^37^; (c) the sensitivity of the systemic agents would be tested in the final surgical specimen so that the adjuvant treatment can be tailored based on the response; (d) clinical trials could be designed on response to therapy as a surrogate of more long-term outcomes.^38^ All of these benefits have replaced the adjuvant with neoadjuvant treatment as the new standard of care in rectal, esophageal, and gastric cancer and the preferred treatment in high-risk breast cancer and soft-tissue sarcoma. In some instances, NAT is done using systemic therapy alone or in conjunction with RT.

The role of NAT for GBM has been explored in phase I/II studies and has yet to be established as a standard of care. To commence a neoadjuvant treatment for GBM, we need a confirmed tissue diagnosis or highly suggestive imaging diagnosis with highly accurate thoughts on the tumor’s molecular profile. These requirements are major challenges in opting for a de-facto NAT for GBM.

First, repeated craniotomy to primarily obtain the tissue samples for a definite diagnosis and identification of the molecular profile and subsequently to resect the residual tumor following NAT would be challenging. Neurosurgical interventions are sophisticated procedures that pose significant operative risks to the patient, namely hemorrhage and infection. To address this concern as an obstacle to NAT, one may propose a stereotactic biopsy to obtain the tissue before commencing treatment.^39^ However, its inherent complexity and need for high-tech devices and skilled neurosurgeons make stereotactic biopsy a nonreadily accessible modality. Thus, this modality should be considered before commencing preoperative therapy to collect samples.

Second, although imaging can accurately define a brain mass lesion as a high-grade glioma, it cannot distinguish GBM from other high-grade gliomas (HGG) or a solitary brain metastasis of an unknown origin.^40^ Considering the improvements in magnetic resonance imaging (MRI), including new sequences and techniques for image acquisition, it is easier to establish a diagnosis of HGG with high accuracy; this may obviate the urgent need to collect tissue for histological diagnosis. Based on its unique imaging characteristics, it is also possible to define the tumor’s molecular profile using advanced MRI techniques, such as apparent diffusion coefficient and relative cerebral blood flow. In addition, major recent developments in the fields of radiomics and radiogenomics have made it possible to predict the response of GBM to specific therapies accurately.^41^ With continuous growth in this field, radiological imaging with new MRI sequences can provide additional tools for improving HGG diagnosis. In cases where biopsy/surgical resection is not feasible, or NAT is planned to be delivered, radiology can also aid in the tumoral profiling of tumors. However, its role is more pronounced in lower-grade gliomas, such as detecting isocitrate dehydrogenase (IDH)-1/2 mutation.^42^ Another useful imaging modality is the positron emission tomography-computed tomography (PET-CT) scan. PET-CT has shown more sensitivity but lower specificity than advanced brain MRI, although the difference was non-significant.^43^ PET-CT with 18F-fluciclovin tracer may find small satellite tumors with a diameter below the usual PET resolution, not noted on MRI.^44^ Although some studies found PET-CT helpful in distinguishing recurrent high-grade glioma from treatment-related changes (pseudoprogression and radionecrosis), further study is needed to address this topic. In general, the role of PET-CT in GBM is investigational.

An alternative for tissue sampling by surgery or biopsy is provided by obtaining a liquid biopsy from plasma or cerebrospinal fluid (CSF). This trending technique offers the advantage of being quicker and less invasive than the conventional brain biopsy while providing valuable diagnostic information. Both plasma- or CSF-based liquid biopsy procedures can identify circulating tumor cells, circulating tumor DNA, and circulating cell-free tumor RNA and discover disrupted signaling pathways to determine the molecular subtype of the tumor, and therefore, the prognosis and response to therapy. Since collecting plasma samples is straightforward for the clinicians and the patients, this method may also be used to monitor the treatment response and to follow-up the patient post-operatively.^45^

Hereunder, we review the published literature on neoadjuvant therapies in 3 different newly diagnosed GBM scenarios: (i) preoperative therapy for GBM; (ii) postoperative neoadjuvant chemotherapy before standard chemoradiation for unresectable or inoperable GBM; and (iii) postoperative neoadjuvant systemic therapy before chemoradiation for resectable GBM;

### Preoperative Neoadjuvant Therapy for GBM

Since the introduction of stereotactic techniques, there has been a renewed interest in studying preoperative RT for primary GBM.^46^ A phase I POBIG trial with awaited results tests the preoperative stereotactic radiosurgery followed by maximal safe resection and standard adjuvant CRT and TMZ.^47^ When it comes to preoperative systemic therapy, we only found 2 studies that looked into the impact of neoadjuvant chemotherapy on surgical results for those with GBM. This is shown in Table 1. The concerns raised and described in the section above might explain the scarcity of literature exploring NAT.

**Table 1.** Summary of Studies Evaluating the Efficacy of Preoperative Therapy for Patients With Newly Diagnosed GBM

Authors	Diagnosis modality	Study type	N. of patients enrolled	Regimen	Studied outcome	Results
Razis et al.^48^	CT-guided biopsy	Phase II study	20	Imatinib 400 mg orally twice daily for 7 days	Cellular markers, OS	Median OS = 6.2 mo Biochemical response to Imatinib: 4/11 of available samples
Miyake et al.^49^	Intra-operative MRI, MET-PET, FMISO-PET, and FLT-PET and 5-ALA	Clinical trial-specify	12	Bevacizumab before surgery vs. surgery alone	Resection rate, PFS, OS	Tumor extraction 97.6% vs. 91.5% by T1-Gd, 95.4% vs. 99.9% by MET, 96.2% vs. 90.2% by FLT, and 97% vs. 92% by FMISO. PFS = 10.1 and 4.9mo, OS = 15.7 and 13.3 mo, respectively

Razis et al. conducted a phase II clinical trial, exploring the efficacy of administration of oral imatinib before definitive surgery for newly diagnosed GBM. The endpoints included overall survival and comparison of the proliferation markers (Ki-67 and CD34) by immunohistochemistry in the pre and postsurgical samples, pharmacokinetics in liquid biopsies, and quantification of imatinib in surgical specimens. All patients underwent a CT-guided biopsy before the start of neoadjuvant imatinib. Median survival was 6.2 months. There was no indication of an impact on proliferation, as demonstrated by the absence of any change in Ki67 expression. The authors concluded that despite the presence of imatinib in the tumor samples and biochemical evidence of response to therapy by changes in activation of AKT and mitogen-activated protein kinase or p27 level in pre- and post-treatment specimens, there was no effect on the patients’ survival nor proliferation of the tumor cells.^48^

In another study, Miyake et al. tested the efficacy of neoadjuvant bevacizumab (BEV) in a phase II randomized clinical trial among GBM patients with low Karnofsky performance status (KPS) or tumors located in eloquent areas.^49^ In this study, the investigators used neuronavigational techniques, including intra-operative MRI, MET-PET, FMISO-PET, FLT-PET, and 5-ALA together with preoperative BEV in 6 out of 12 patients. Using BEV made the resection time shorter, and the residual volume smaller in a way that more patients had >95% resection rate in the BEV group. The authors concluded that BEV improved patients’ prognosis and outcomes in a preoperative setting.

Although no definite conclusions can be drawn based on these 2 trials, de-facto NAT may be a new promising approach for a subset of GBM patients with low KPS. At the same time, further research is needed to create more solid evidence. Actually, the study of preoperative therapies is strongly limited by the need for surgical diagnostic specimens. Thus, efforts should be focused on improving the radiological tools for non-surgical diagnoses in the future.^50^

Nevertheless, the expected benefit of BEV is not correlated with MGMT status.^51^ However, the usual benefit from IO or TKIs usually correlates with some molecular alterations in the tumoral tissue.

### Postoperative Neoadjuvant Systemic Therapy Before Standard Chemoradiation for Unresectable or Inoperable GBM

As described earlier, surgical resection constitutes an integral part of GBM treatment together with RT and systemic therapy with or without TTF. However, surgery may be limited by the presence of various factors: (i) patient-related such as low KPS score, advanced age, medical unfitness for surgery, or (ii) tumor-related such as multifocality, tumors localized in eloquent areas where resection is not feasible. In these cases, tumor diagnosis is based on stereotactic biopsy or neuroimaging. The overall prognosis in unresected tumors is dismal.^52^ The management includes RT, with or without concurrent and sequential chemotherapy or chemotherapy alone. In the case of bulky tumors, the target volume for RT due to its large size may cause increased side effects, in turn leading to decreased RT compliance. In addition, the necrotic inherence of extensive GBM tumors with severe hypo-oxygenation of tumor cells confers higher radioresistance, making the efficacy of irradiation more limited when compared to smaller-size tumors.^53^ These limitations have led to the pursuit of neoadjuvant systemic therapy before the RT delivery. The rationale of this approach is that after the damage to the BBB, the anti-tumor agent can penetrate and concentrate more in the tumor and be more effective.^54^ Besides, delivering systemic agents with the ability to cross the BBB in a neoadjuvant setting offers the potential advantage of the tumor to shrink, thus decreasing the RT target volume with the benefit of less acute and late side effects. This reduction in volume may result in improved neurological symptoms after RT completion and, subsequently, patients’ performance status. The latter is especially important for methylated MGMT cases with a better prognosis when treated with standard RT and concurrent TMZ.

On the contrary, initiating RT as soon as possible may be clinically beneficial to the patients by inducing tumor shrinkage in unresectable GBM and improving associated neurological symptoms. The true benefit of NAT has not been tested in a randomized setting and may prolong the median time from surgery to RT initiation. Nevertheless, Balaña et al. showed that prolongation caused by NAT was not an adverse prognostic factor for OS and that patients who started RT after the optimal cut-off duration of 6.43 weeks had a longer OS compared to those who began RT before this point (median 19.1 months vs. 6.6 months, *P* = .005)^55^; therefore, the longer diagnosis-to-RT interval for delivering neoadjuvant chemotherapy might be not hazardous.

Studies investigating the benefits and toxicity of neoadjuvant postoperative systemic therapy before CRT as the primary adjuvant treatment using various regimens are represented in Table 2.

**Table 2.** Summary of Studies Evaluating the Efficacy of Neoadjuvant Chemotherapy Before Chemoradiation for Patients With Unresectable Newly Diagnosed GBM

Authors	N. of patients	Regimen	Control group	Studied outcomes	Results	Conclusions
Barrie et al.^56^	40	Carmustine (150 mg/m^2^) on day 1, TMZ (110 mg/m^2^/day) on days 1–5 every 42 days for 4 cycles before RT, followed by the same regimen after RT until disease progression or unacceptable toxicity	None	ORR, OS, and PFS	ORR = 42.5% (5% complete and 37.5% partial response), 24% stable and 35% progressive disease, median OS = 12.7 mo, median PFS = 7.4 mo	
Chinot et al.^57^	29	TMZ (150 mg/m^2^/day) on days 1–7 and 15–21 every 28 days (7 days on/7 days off) before RT	None	RR, OS, and PFS	Overall, 24% had partial response, 31% were stable, and 41% had progressive disease, with median PFS = 3.8 mo and median OS = 6.1 mo. High response rate (55%), PFS (5.5 mo), and OS (16 mo) in patients with MGMT methylation	Modest efficacy of neoadjuvant dose-dense TMZ but inferior to standard concomitant chemoradiotherapy in the whole group of patients
Bihan et al.^58^	8	BEV (10 mg/kg) every 2 weeks before systemic chemotherapy (either TMZ 150 mg/m^2^/day for 5 days) every month or fotemustine (80 mg/m^2^ every 2 weeks), then RT	None	Clinical condition, OS	Clinical improvement in all patients: 4 became stable and had a prolonged survival.	Suggest the benefit of neoadjuvant, which needs confirmation.
Lou et al.^59^	41	TMZ (200 mg/m^2^/day) on days 1–5 for 4 cycles, BEV (10 mg/kg) on days 1, 15	None	RR evaluated by MRI, toxicity	24.4% partial response, 68.3% stable disease, 2.4% progression	Should be investigated in phase III trials
Chauffert et al.^60^	120	BEV (10 mg/kg) + IRI (125 mg/ m^2^) every 2 weeks for 4 cycles before RT with TMZ (75 mg/m^2^/day) + BEV every 2 weeks and adjuvant BEV + IRI every 2 weeks for 6 mo	Use of BEV at progression	OS and toxicity	Only 30/90 were alive without progression after 6 months, PFS = 7.1 mo in case vs. 5.2 mo in control, OS was similar.	Neoadjuvant and adjuvant BEV/IRI are not recommended when combined with TMZ-RT.
Capdevila et al.^30^	46	Two cycles of neoadjuvant TMZ + cisplatin before RT	RT + TMZ, followed by adjuvant TMZ	RR, OS, PFS	OS and PFS were 8.5 and 3.3 mo in group 1; 11.2 and 5.1 mo in group 2	Neoadjuvant TMZ + cisplatin did not affect patients’ outcome
Peters et al.^61^	41	Upfront TMZ (200 mg/m^2^/day) on days 1–5 for 4 cycles, BEV (10 mg/kg), and IRI every 2 weeks until disease progression before chemoradiotherapy	None	RR, OS, PFS	22% partial response, 61% stable, and 4.9% progression. RR = 22%, median OS = 12 mo	Upfront BEV, TMZ, and IRI are tolerable with favorable RR
Balaña et al.^62^	102	TMZ + BEV (10 mg/kg) on days 1–15 of each cycle and days 1, 15, and 30 of combination therapy with RT	TMZ (85 mg/m^2^/day) on days 1–21 (2 28–day cycles), TMZ + RT, and 6 adjuvant TMZ cycles	RR, OS, and PFS	Higher objective response and more toxicity rate in TMZ + BEV, no difference in OS or PFS	TMZ + BEV is a more active neoadjuvant therapy but with greater toxicity
Chaskis et al. ^63^	12	TMZ 150 mg/m^2^/day for one 5-day course before RT with CON and ADJ TMZ	None	PFS and OS	PFS and OS were 90% and 91.7% at 6 months, 58.3% and 71.3% at 12 months, 31.1% and 71.3% at 18 months, respectively.	Toxicity and OS in this small study with poor prognosis patients were similar to large studies with the general population with GBM.

BEV: bevacizumab; IRI: irinotecan; TMZ: temozolomide; OS: overall survival; PFS: progression-free survival: RR: response rate; ORR: objective response rate; CON: concomitant; ADJ: adjuvant.

Barrie et al. evaluated the role of TMZ plus carmustine (BCNU) before and after RT in unresectable GBM patients. They showed that this regimen is safe and effective. The median OS was 12.7 months, and the median PFS was 7.4 months. Forty-two percent of patients had a partial or complete response, similar to the response rate of neoadjuvant TMZ only in sub-totally resected GBM patients.^56^

In the study by Chinot et al., biopsied-only patients with GBM received 4 cycles of TMZ alone (150 mg/m^2^/d on days 1–7 and on days 15–21 of every 28-day cycle (7 days on/7 days off)), before and after RT (60 Gy in 30 fractions). The objective response rate in patients with low MGMT methylation vs. high MGMT methylation was 55% vs. 7%, respectively (*P* = .004). High expression was defined as more than 35% of tumor cell nuclei expressing detectable MGMT protein; low expression was defined as less than 35%. PFS and OS were prolonged by 3.6 months (*P* = .009) and 11 months (*P* = .003), respectively, in patients with low vs. high MGMT expression. However, this result may be confounded by differences in the extent of RT in these subgroups. The authors concluded that neoadjuvant chemotherapy may be feasible for inoperable GBM patients with low MGMT methylation status.^57^

Bihan et al. retrospectively evaluated the efficacy of BEV plus TMZ or fotemustine in poor-performance status patients with unresectable GBM as a bridge to standard chemoradiotherapy. Neoadjuvant treatment was delivered until maximal clinical and radiological response. The median OS was 12.5 months which was unusually high for these poor-performance patients. They concluded that rapid use of BEV plus TMZ helped improve the delivery of RT to a smaller area with a standard dose while sparing more unaffected brain tissue.^58^

In the study of Lou et al., patients received TMZ plus BEV before standard RT dose with concurrent TMZ + BEV. Median survival was 11.7 months. This combination was generally tolerable, though one potential study-related death was reported due to myocardial infarction (G5 toxicity). The authors concluded that TMZ and BEV in the neoadjuvant setting stabilized the gross disease in multifocal and unresectable GBM cases and merits further well-designed phase III trials.^59^

Chauffert et al. initiated a randomized phase II trial evaluating neoadjuvant BEV plus irinotecan (IRI) before CRT vs. adjuvant BEV and IRI. Despite higher 6-month PFS rates (50% vs. 30%), the OS was the same in both arms (median OS = 11.1 months). They concluded that the combination of neo-adjuvant and adjuvant BEV with IRI, TMZ, and RT is not recommended for further evaluation in the first-line treatment of unresectable GBM.^60^

Capdevila et al. compared the results of 2 consecutive cohorts with unresectable GBM patients. The first cohort used 2 cycles of neoadjuvant chemotherapy (cisplatin plus TMZ) prior to local irradiation. In the second cohort, the established current standard CRT was done. Median PFS was not different between the 2 cohorts (3.3 vs. 5.1 months, *P* = NS). However, patients with methylated MGMT had a better outcome regarding PFS and OS with the neoadjuvant chemotherapy. Patients without MGMT methylation showed a better outcome regarding PFS and OS with upfront standard CRT. The authors concluded that neoadjuvant TMZ plus cisplatin has no positive or negative effect on the outcome of patients with unresectable GBM or anaplastic astrocytoma. Besides, adding cisplatin to the neoadjuvant regimen does not increase the benefit obtained from TMZ alone. They also highlighted the importance and possibility of integrating molecular testing into treatment planning.^30^

In the study by Peters et al., patients were treated with TMZ + BEV + IRI. Only 4.9% showed tumor progression. Median overall survival was 12 months, and median progression-free survival was 8.6 months (95% CI: 3.5–11.3 months). They concluded that upfront treatment with BEV, TMZ, and IRI is tolerable and can lead to a radiographic response in unresectable or subtotally resected GBM.^61^

Balana et al. compared neoadjuvant and concurrent TMZ with TMZ + BEV. Their primary endpoint was response assessment. The investigational treatment was more active than TMZ alone at the expense of greater toxicity. In the TMZ arm, the leading cause of NAT discontinuation was disease progression. In contrast, in the investigational arm, it was toxicity. Patients in the TMZ + BEV arm experienced more grade 1–2 stomatitis. Clinically significant intracranial hemorrhage occurred in 4 patients in the combination arm, including 2 deaths. A third patient in the TMZ + BEV arm died from intestinal perforation. There was no other significant difference in the frequency of toxicities between the two components. Although more patients suffered from toxicity in the combination arm, more patients completed the treatment, and neurological decline was more common in the TMZ arm. Eleven patients (24.5 %) in the TMZ arm and 29 (60.4 %) in the TMZ + BEV arm attained clinical benefit (partial response or stable disease). There was a trend toward better PFS (4.8 vs. 2.2 months) and OS (10.6 vs. 7.7 months) in the investigational arm. Although this study was not powered to detect differences in PFS or OS, it reached its primary endpoint of higher response. As in the TEMAVIR study, they found more intracranial hemorrhage in the BEV arm (4.2 % vs. none in the control arm).^62^

As described above, neoadjuvant chemotherapy in the biopsied-only GBM patients showed inconclusive results. This failure may be owed to administering different neoadjuvant regimens and variable control groups. A well-designed randomized phase III trial is necessary to generate solid evidence with more robust conclusions—for instance, one comparing neoadjuvant TMZ ± BEV with the current standard of care. Integrating the MGMT status in the trial design is highly recommended to stratify the effect on treatment response and survival.

### Postoperative Neoadjuvant Systemic Therapy Before Chemoradiation for Resectable GBM

It is well established that GBM represents an aggressive disease with a doubling time of 49.6 days.^63^ Therefore, given that time is essential, CRT after surgery as the standard of care has the disadvantage of allowing the tumor to develop and progress during the gap between surgery and RT start.^64^ This interval is influenced by several factors, including the availability of histopathological results, limited access to RT facilities, a waiting list for RT start, and post-op imaging uncertainties to define target volume for an accurate RT delivery. These factors all can result in a negative impact on the patient’s prognosis.^65^ This concern justifies the use of neoadjuvant systemic treatment for patients with newly diagnosed GBM. Using this treatment option, we can limit tumor progression, optimize the extent of tumor resection, and improve the patient’s prognosis.

A summary of clinical trials investigating the efficacy of chemotherapy before CRT for patients with newly diagnosed resectable GBM is represented in Table 3. Other studies evaluating the effect of neoadjuvant chemotherapy on different types of glioma, such as low-grade glioma,^75,76^ or studies that did not isolate GBM when evaluating HGG^77^ were not included in this table, as we aimed only to review the treatment options for GBM.

**Table 3.** Summary of Studies Evaluating the Efficacy of Chemotherapy Following Surgery Before Chemoradiation for Patients With Newly Diagnosed Resectable GBM

Authors	Study type	No. of patients	Intervention group	Control group	Studied outcomes	Results	Conclusions
Gruber et al.^66^	Phase II clinical trial	25 (10 GTR)	Four cycles of carboplatin 600 mg/m^2^ once every 4 weeks before RT	None	OS, RR, complications	1 CR and 2 PR; 7 SD, 1 PD, OS = 19.2 mo	Feasibility of neoadjuvant chemotherapy
Gilbert et al.^67^	Phase II clinical trial	36 (33 adult, 3 pediatric)	Max. 4 cycles of TMZ 200 mg/m^2^/day for 5 days before RT	None	RR, OS, PFS,	11% CR and 31% PR, 28% SD, OS = 13.2 and PFS = 3.9mo	Safe and well-tolerated
Choi et al.^68^	Phase II clinical trial	30	Two cycles of 40 mg/m^2^/day nimustine + cisplatin before RT	None	OS, RR, complications	5% CR and 36% PR, 14% SD, 45% PD, OS = 14.9mo	Pre-irradiation chemotherapy is effective and feasible
Kim et al. 2011^69^	Phase III trial	82	Neoadjuvant nimustine (ACNU) 40 mg/m^2^/day + cisplatin 40 mg/m^2^/day for 2 cycles 6 weeks apart, then RT followed by 6 cycles of TMZ for 5 days.	RT followed by 6 cycles of TMZ for 5 days after surgery	OS	Interventions vs. control Median OS = 28.4 vs. 18.9 mo; 2-year OS was 50.9% vs. 27.8%, PFS = 6.6 vs. 5.1 mo	Despite auspicious outcomes, the intervention’s high rate of severe hematologic toxicity limits its use
Wick et al.^70^	Phase II clinical trial	60	Oral enzastaurin 500 mg once daily (QD) or 250 mg twice daily (b.i.d.) concurrent RT (1.8- to 2.0-Gy) 5 days/week for 6 weeks	EORTC 26981/22981 NCIC CE.3 trial	PSF-6, OS	PFS-6 = 53.6%, median OS = 15 mo	The safety profile was as expected as the previous trial, well-tolerated
Hofland et al.^71^	Phase II randomized trial	65	BEV + IRI for 8 weeks before RT plus BEV + IRI, followed by another 8 weeks.	BEV + TMZ for 8 weeks before RT plus BEV + TMZ, followed by another 8 weeks.	RR, PFS, toxicity	RR = 32% for control and 23% for case group (*P* = .56), median PFS = 7.7 and 7.3 mo, hematologic toxicity in BEV + TMZ	Neoadjuvant BEV + TMZ was superior to BEV + IRI
Mao et al.,^72^	Phase II randomized trial	99	TMZ 75 mg/m^2^/day for 2-wks followed by concomitant TMZ + RT and ADJ TMZ	Concomitant TMZ + RT and ADJ TMZ	PFS, OS	OS = 17.6 vs. 13.2 (*P* = .021) PFS = 8.7 vs. 10.4 (*P* = .695)	Prolonged OS by neoadjuvant TMZ
Shenouda et al.^73^	Phase II clinical trial	50	TMZ 75 mg/m^2^/day for 2 weeks before RT	None	OS, PFS, toxicity	Median OS = 22.3 mo, PFS = 13.7 mo, 4-year OS = 30.4%	Favorable long-term survival
Jiang et al. 2019	Retrospective	375 (163 NEO, 212 ADJ)	Super-early initiation of TMZ within 7 days after craniotomy followed by Stupp Protocol	Standard Stupp Protocol	OS, PFS	OS = 23 vs. 17 mo HR = 0.583, 95% CI 0.384–0.884, *P* = .011 PFS = 11.5 vs. 9 mo (NS)	Super-early initiation of TMZ may confer survival benefits, especially for those without GTR or methylated MGMT (even PS is significant)

GTR: gross-total resection; PR: partial response; CR: complete response; SD: stable disease; PD: progressive disease; BEV: bevacizumab; IRI: irinotecan; TMZ: temozolomide; OS: overall survival; PFS: progression-free survival; RR: response rate.

First, Gruber et al. evaluated the efficacy and toxicity of postop neoadjuvant carboplatin in 25 patients. They demonstrated the feasibility and modest toxicity of the regimen.^78^ Gilbert et al. evaluated TMZ before RT alone. The objective response to TMZ treatment was 39%. In addition, 32% of patients had stable disease. They concluded that postop neoadjuvant TMZ is well tolerated and probably as effective as other more toxic chemotherapy agents like nitrosourea or cisplatin. They also proposed an evaluation of this regimen with CRT with TMZ in upcoming trials.^66^ Choi et al. evaluated ACNU and cisplatin and found that it is feasible to administer an intensive chemotherapy regimen to these patients. They saw that although the prognosis may be worse in patients with tumors growing during chemotherapy, some would stabilize after RT.^67^

Kim et al. performed a prospective randomized controlled multicenter phase III trial to evaluate 2 cycles of neoadjuvant ACNU plus cisplatin (CDDP), followed by standard conventional radiotherapy followed by 6 cycles of adjuvant temozolomide as in the control group. The study was closed after an interim analysis of 82 patients due to a high frequency of toxic side effects despite promising actuarial survival outcomes. Although the median survival benefit in the treatment group over the control group was 9.5 months, this difference did not reach statistical significance (*P* = .2). Similarly, the progression-free survival was not different. Importantly, meta-analyses have confirmed the anti-cancer effects of nitrosourea compounds, including survival benefits. The authors concluded that neoadjuvant chemotherapy with ACNU-CDDP followed by radiotherapy and adjuvant temozolomide as primary treatment for GBM^68^ So, the upcoming trials skipped using ACNU or CDDP.

Hodfland et al. randomized 65 patients to bevacizumab-irinotecan (Bev-Iri) or bevacizumab-temozolomide (Bev-Tem) for 8 weeks, followed by concomitant CRT and adjuvant chemotherapy with Bev-Iri or Bev-Tem. They concluded that irinotecan has no benefit in first-line therapy compared to temozolomide. Moreover, only the Bev-Tem arm met the prespecified activity level of interest.^69^

Mao et al. compared the standard TMZ regimen plus early post-surgery TMZ (early TMZ group) vs. the standard TMZ regimen (control group). Median OS time was 17.6 months in the early TMZ group and 13.2 months in the control group (*P* = .021). They mentioned that adding 2 weeks of TMZ starting the 14th day after surgery to the standard regimen can improve OS in GBM patients with similar AE and SAE occurrences.^70^ Shenouda et al. evaluated neo-adjuvant TMZ started 2–3 weeks following surgery at a daily dose of 75 mg/m^2^ for 2 weeks before delivering HART (60 Gy in 20 daily fractions) with concurrent and adjuvant TMZ in 50 patients. Their study showed that this novel approach of neo-adjuvant TMZ is associated with encouraging favorable long-term survival with acceptable toxicity. A future comparative trial of the efficacy of this regimen is warranted.^71^

Jiang et al. retrospectively evaluated 375 patients with GBM. One hundred sixty-three patients received super-early TMZ within 7 days (SEG), while 212 received conventional protocol alone (CG). Due to the retrospective nature of the present study, they conducted propensity score matching to reduce the bias in patient selection and aimed to disclose the actual effect of the super-early initiation of TMZ. Final results showed that the median OS of patients in SEG was significantly prolonged without additional adverse effects compared with those in CG. This survival benefit was more prominent in patients with MGMT promoter methylation or non-GTR than vice versa. The adjusted hazard ratio for death in SEG was 0.60, indicating a 40% relative reduction in the risk of death for patients treated with super-early TMZ. Thus, they mentioned that perhaps 1 week after surgery is the ideal time window to initiate TMZ, especially for those with MGMT promoter methylation or non-GTR.^72^

As mentioned above, a few small sample-size studies have evaluated the efficacy and safety of neoadjuvant chemotherapy for patients with newly diagnosed GBM. The ORR varied between 12% and 42%; a stable disease was reported in 14–28% of cases. The median PFS and OS ranged from 3.9 and 13.2 months to 13.7 and 22.3 months, respectively. Again, as different treatment regimens have been applied in these studies, it is more complex to draw definite conclusions. Most studies reported longer survival rates with the proposed interventions than the standard treatment in actual settings.

Nevertheless, the most effective and less toxic regimens include TMZ and BEV, administered together or alone. Based on the last 2 studies, neoadjuvant pre-radiation chemotherapy is most effective when started within the first 2 weeks after surgery. An ongoing large-scale multicenter study called MAGMA is testing the idea of the benefit of neoadjuvant chemotherapy before chemoradiotherapy vs. extended adjuvant therapy in newly diagnosed GBM.^73^ The results of this study help provide a clearer picture of neoadjuvant chemotherapy.

## Conclusions and Future Perspectives

In this review article, most studies on neoadjuvant treatment for newly diagnosed GBM were reviewed. Although in the latest WHO classification (2021) for brain tumors, IDH-wildtype astrocytoma (formerly classified as a lower grade diffuse glioma) is molecularly and prognostically considered in the same category as GBM, this subgroup was not included in the current review. Given that most previous studies have not outlined the IDH mutation status, it is impossible to evaluate the outcomes from other HGG and reach a definite conclusion.^74^ In some of these studies, no effective neoadjuvant treatment including preoperative CRT was used for GBM. Some studies did not perform surgery, which is the cornerstone of GBM management; some only prescribed postoperative chemotherapy before standard adjuvant CRT, and others only used chemotherapy before surgery. Also, the only de facto preoperative CRT trial has not been published yet (PARADIGMA Trial ClinicalTrials.gov Identifier: NCT03480867). Despite these pitfalls in examining neoadjuvant regimens, promising results have been identified. It is possible to draw 2 conclusions. First, the most frequently used chemotherapy regimen was TMZ plus bevacizumab, which had a promising impact on the outcomes. The merits should be evaluated in a well-designed study for further conclusions. Second, completion of RT had a significant impact on survival. It should be noted that most patients in these trials were not eligible for the standard multimodality treatment, as some had morbidities that prevented surgery, others a low KPS, and extensive unresectable tumors.

Considering the improvements in radiomics, radiogenomics, and liquid biopsy for unresectable or borderline resectable gliomas, more robust recommendations can be proposed regarding selecting candidates for NAT. We suggest exploring the use of CRT with a standard dose of TMZ in the forthcoming trials, besides standard TMZ-based chemotherapy as a postoperative pre-radiotherapy treatment. To examine the use of preoperative chemotherapy alone, we recommend considering TMZ + bevacizumab for 2–3 months as a viable option. This chemotherapy can be followed by standard CRT for adjuvant treatment of any residual tumors. We suggest testing this approach in future trials to determine its effectiveness. To accurately assess the effects of NAT, following the current standard of administering full-dose MGMT-tailored adjuvant treatment is necessary.

Using targeted agents and immunotherapy could have been more helpful for selected cases. However, a small subgroup of patients may benefit significantly, especially from immune checkpoint inhibitors.^28^ We suggest a strict patient selection for forthcoming trials involving immunotherapy in primary or recurrent tumors as we learned from other solid tumors that not all patients are well-responders, and toxicity may be high.^79^ However, ICIs could be combined with OV-based therapies to see if heating the immunogenic environment of GBM could improve the efficacy of ICI.
